# Supplementary figures and images for: The causal effects of circulating cytokines on sepsis: a Mendelian randomization study
Source: PeerJ. 2024 Feb 1;12:e16860. doi: 10.7717/peerj.16860 (PMC10838533; doi:10.7717/peerj.16860)

# MR Test

- Inverse variance weighted
- MR Egger
- Simple mode
- Weighted median
- Weighted mode

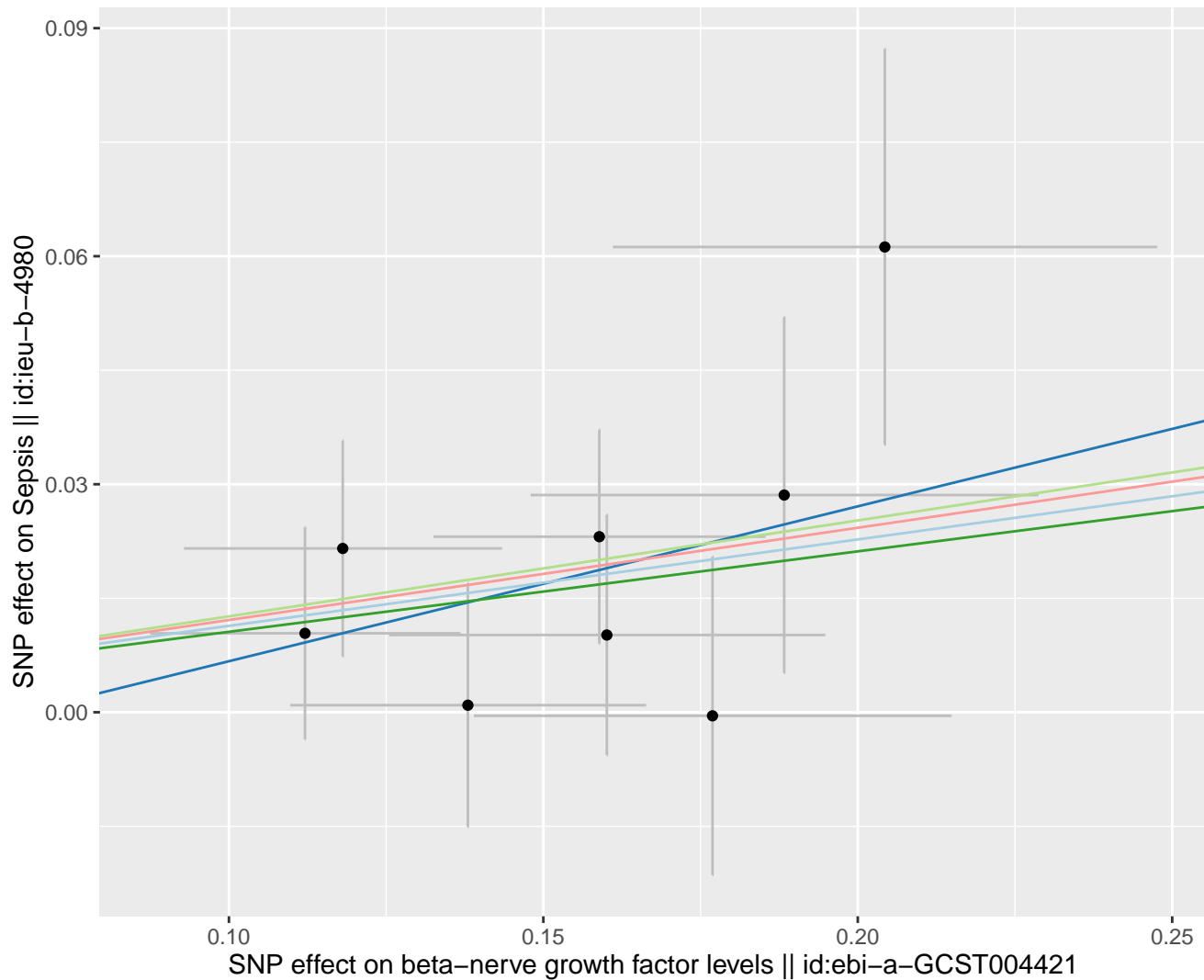

Supplement: Supplemental Information 1 [file peerj-12-16860-s001.pdf]

# MR Test

- Inverse variance weighted
- MR Egger
- Simple mode
- Weighted median
- Weighted mode

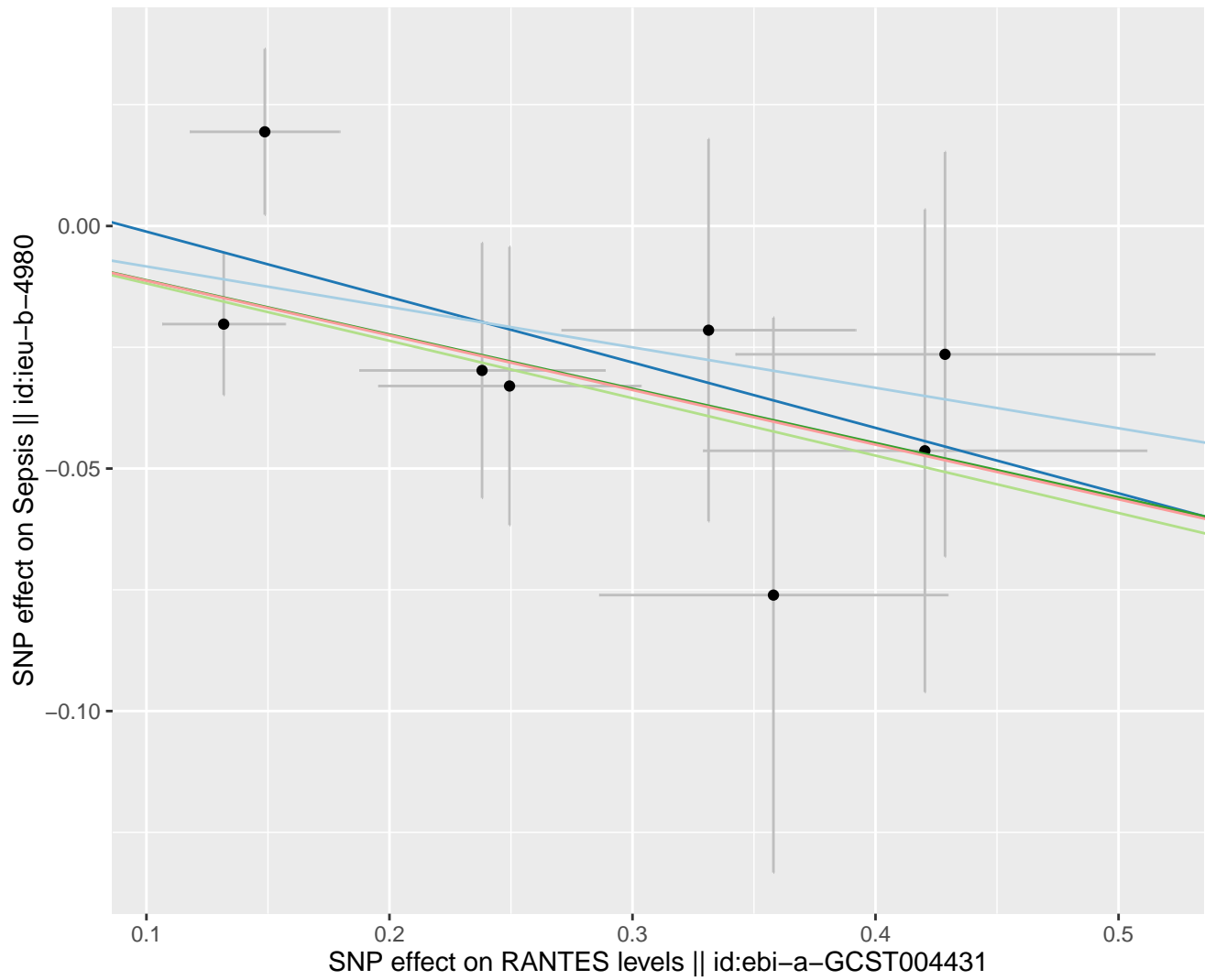

Supplement: Supplemental Information 3 [file peerj-12-16860-s003.pdf]

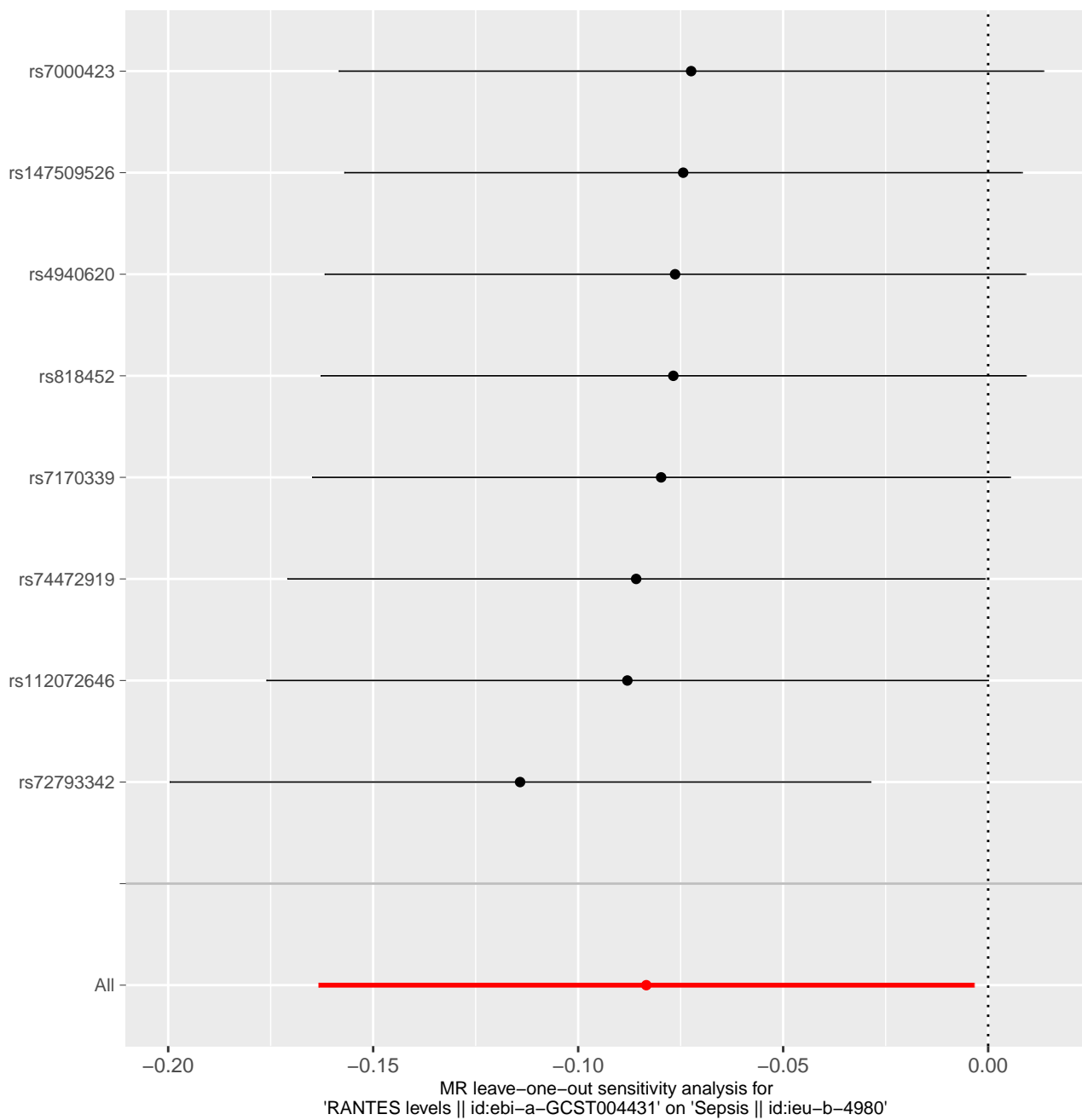

Supplement: Supplemental Information 4 [file peerj-12-16860-s004.pdf]

# MR Test

- Inverse variance weighted
- MR Egger
- Simple mode
- Weighted median
- Weighted mode

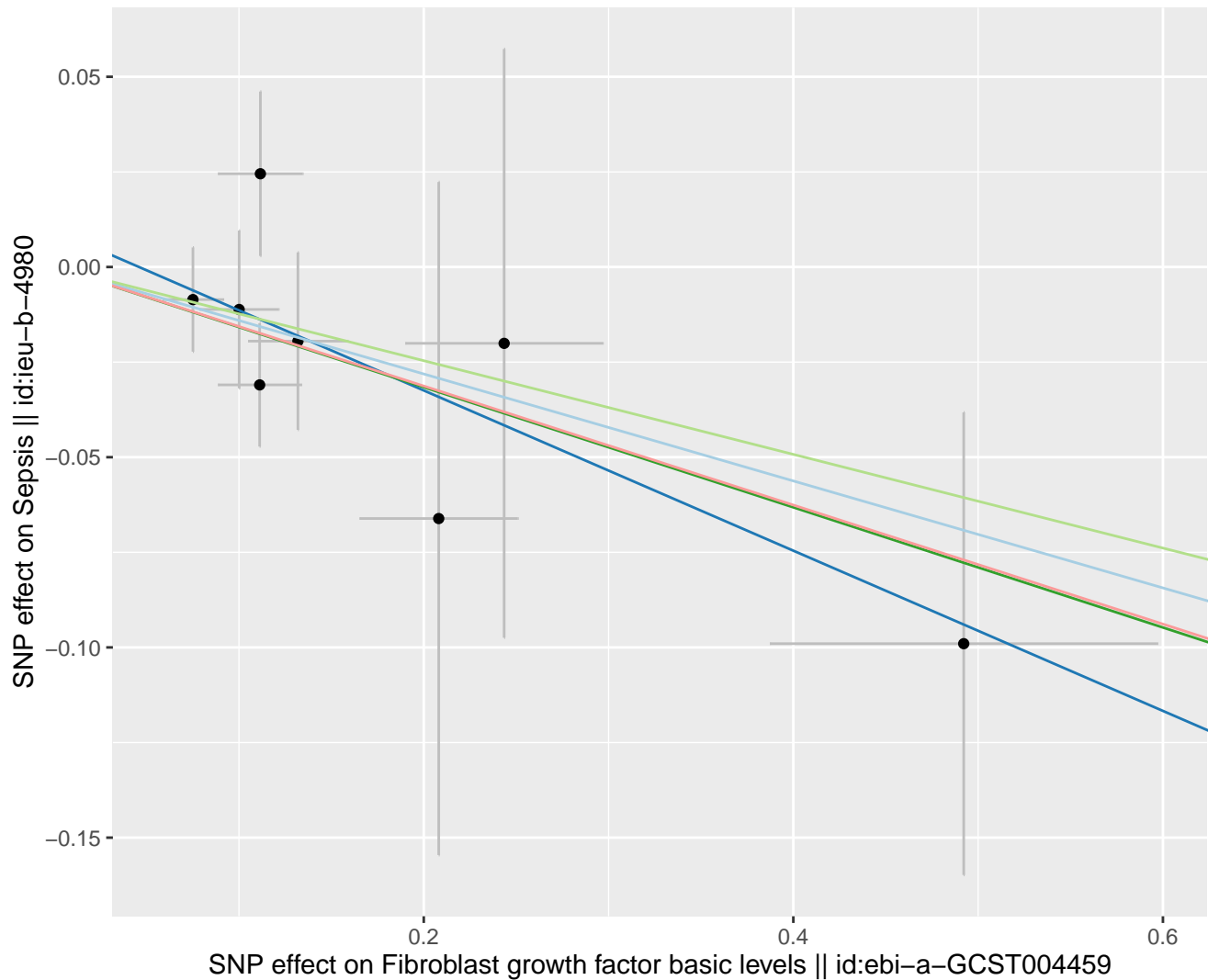

Supplement: Supplemental Information 5 [file peerj-12-16860-s005.pdf]

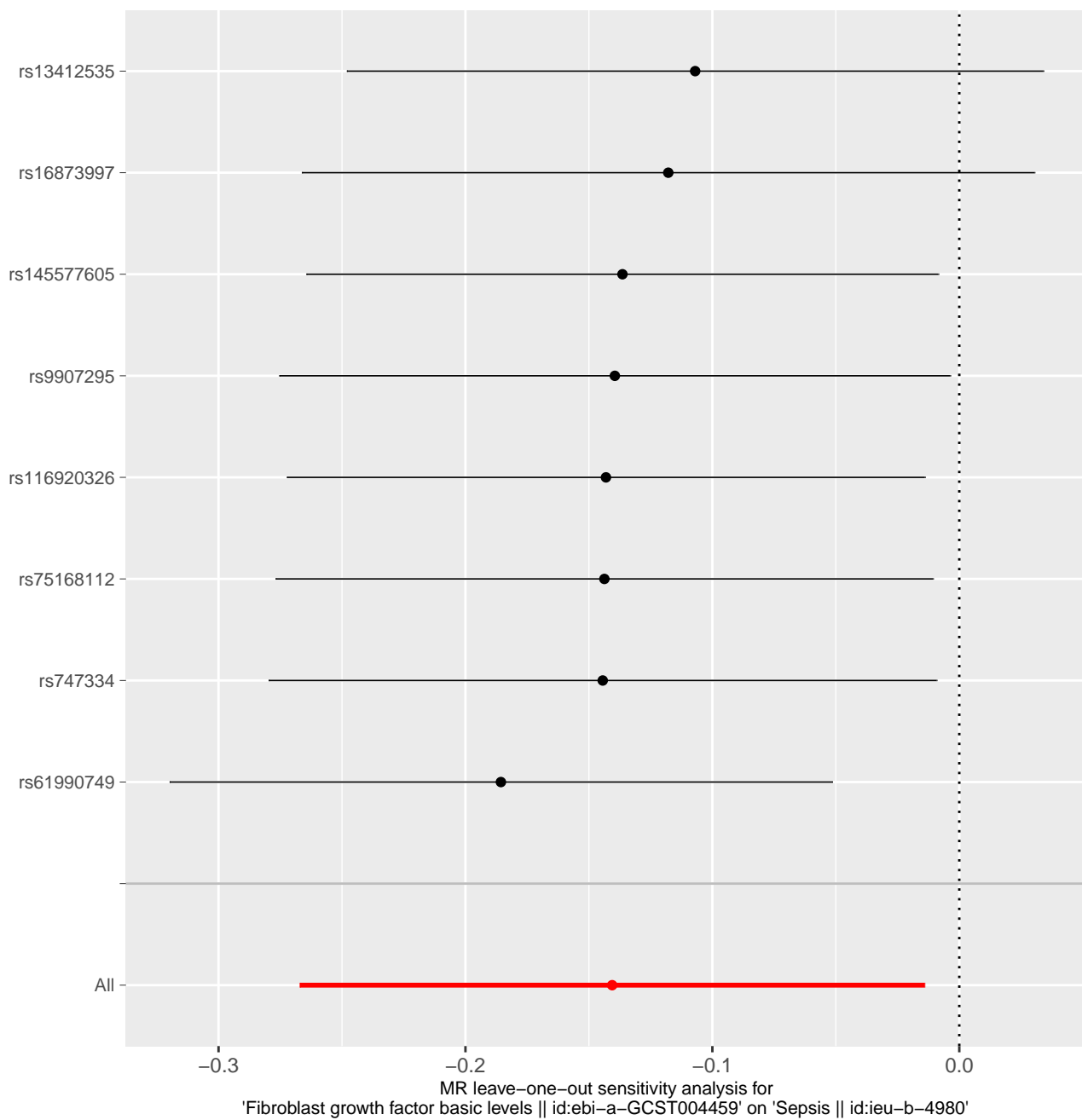

Supplement: Supplemental Information 6 [file peerj-12-16860-s006.pdf]
