# Supplementary material for: The causal effects of circulating cytokines on sepsis: a Mendelian randomization study
Source: PeerJ. 2024 Feb 1;12:e16860. doi: 10.7717/peerj.16860 (PMC10838533; doi:10.7717/peerj.16860)
Supplement: Supplemental Information 2 [file peerj-12-16860-s002.pdf]

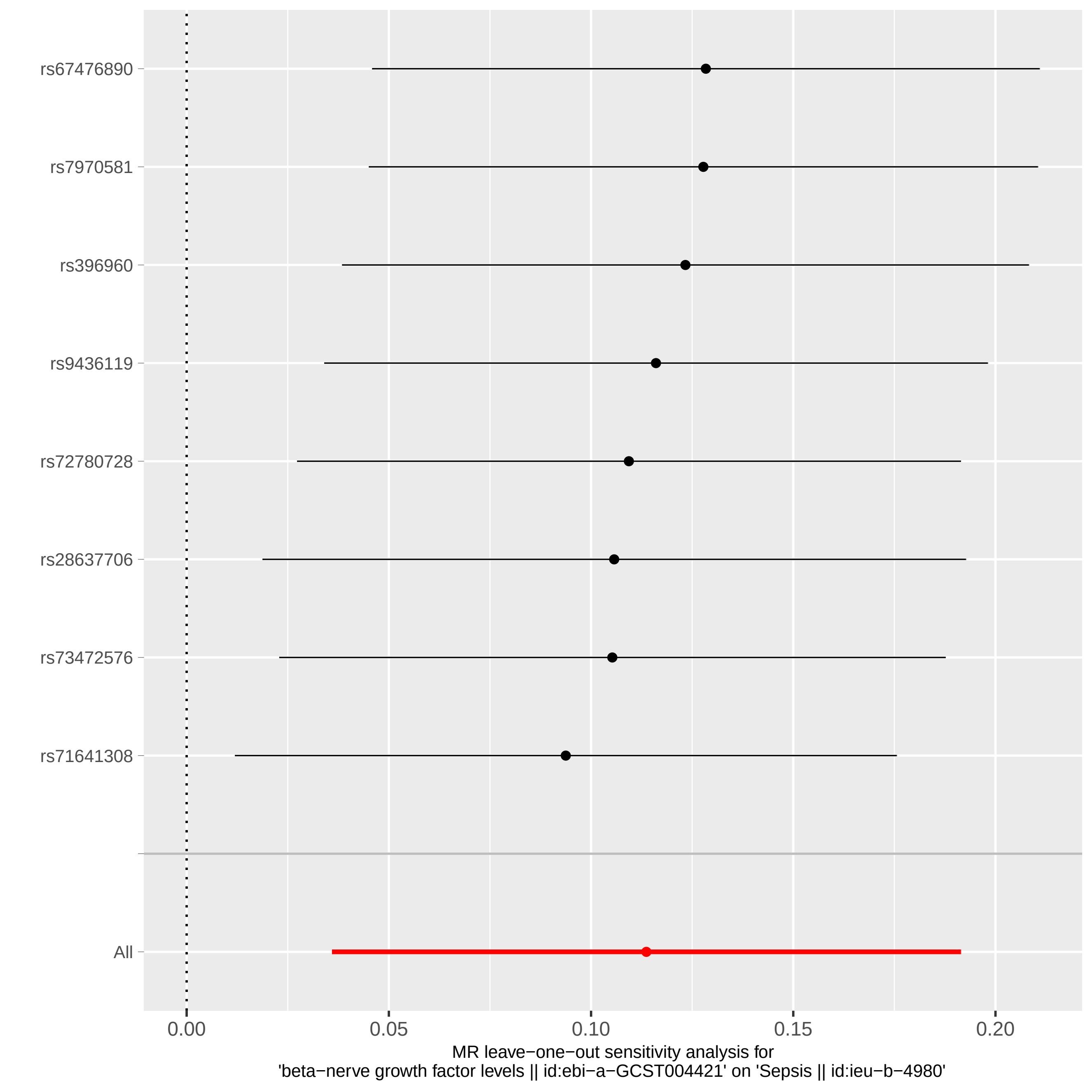

rs67476890
rs7970581
rs396960
rs9436119
rs72780728
rs28637706
rs73472576
rs71641308
All
0.00
0.05
0.10
0.15
0.20
MR leave−one−out sensitivity analysis for
'beta−nerve growth factor levels || id:ebi−a−GCST004421' on 'Sepsis || id:ieu−b−4980'
