# Supplementary material for: The causal effects of circulating cytokines on sepsis: a Mendelian randomization study
Source: PeerJ. 2024 Feb 1;12:e16860. doi: 10.7717/peerj.16860 (PMC10838533; doi:10.7717/peerj.16860)
Supplement: Supplemental Information 10 [file peerj-12-16860-s010.docx]

**STROBE-MR checklist of recommended items to address in reports of Mendelian randomization studies**^1^ ^2^

| **Item No.** | **Section** | **Checklist item** | **Page No.** | **Relevant text from manuscript** |
| --- | --- | --- | --- | --- |
| 1 | **TITLE and ABSTRACT** | Indicate Mendelian randomization (MR) as the study’s design in the title and/or the abstract if that is a main purpose of the study | 1 | The casual effects of circulating cytokines on sepsis: a mendelian randomization study |
|  | **INTRODUCTION** |  |  |  |
| 2 | **Background** | Explain the scientific background and rationale for the reported study. What is the exposure? Is a potential causal relationship between exposure and outcome plausible? Justify why MR is a helpful method to address the study question | 1 | Sepsis and circulating cytokines levels have been associated in observational studies with unclear causality. |
| 3 | **Objectives** | State specific objectives clearly, including pre-specified causal hypotheses (if any). State that MR is a method that, under specific assumptions, intends to estimate causal effects | 1 | Mendelian randomization (MR) was used to identify the causal direction between circulating cytokines and sepsis in a two-sample study. |
|  | **METHODS** |  |  |  |
| 4 | **Study design and data sources** | Present key elements of the study design early in the article. Consider including a table listing sources of data for all phases of the study. For each data source contributing to the analysis, describe the following: | 2 | Study design  Genome-Wide Association Summary Data |
|  | a) | Setting: Describe the study design and the underlying population, if possible. Describe the setting, locations, and relevant dates, including periods of recruitment, exposure, follow-up, and data collection, when available. | 2 | The Cardiovascular Risk in Young Finns study, FINRISK 1997, and FINRISK 2002 provided summary-level data for 41 cytokines  UK Biobank provided summary-level data of GWAS for sepsis, which included 10,154 cases and 452,764 controls |
|  | b) | Participants: Give the eligibility criteria, and the sources and methods of selection of participants. Report the sample size, and whether any power or sample size calculations were carried out prior to the main analysis | 2 | UK Biobank provided summary-level data of GWAS for sepsis, which included 10,154 cases and 452,764 controls  And female constitute 54%. The median age of all participants was 58 years, and the median age of those suffering from sepsis was 60 years. |
|  | c) | Describe measurement, quality control and selection of genetic variants | 2 | In order to obtain reliable findings, this study was based on three major assumptions underlying MR studies. First, instrumental variables (IVs) must be strongly correlated with circulating cytokines. Second, instrumental variables are independent of confounding factors. Third, instrumental variables act on sepsis through circulating cytokines |
|  | d) | For each exposure, outcome, and other relevant variables, describe methods of assessment and diagnostic criteria for diseases | 2 | Global Burden of Disease (GBD) study codes were used to define sepsis based on International Classification of Disease (ICD)-9 and ICD-10 codes Study design  Genome-Wide Association Summary Data |
|  | e) | Provide details of ethics committee approval and participant informed consent, if relevant | NA | NA |
| 5 | **Assumptions** | Explicitly state the three core IV assumptions for the main analysis (relevance, independence and exclusion restriction) as well assumptions for any additional or sensitivity analysis | 2 | In order to obtain reliable findings, this study was based on three major assumptions underlying MR studies. First, instrumental variables (IVs) must be strongly correlated with circulating cytokines. Second, instrumental variables are independent of confounding factors. Third, instrumental variables act on sepsis through circulating cytokines |
| 6 | **Statistical methods: main analysis** | Describe statistical methods and statistics used | 2-4 | Selection of IVs  Statistical Analyses |
|  | a) | Describe how quantitative variables were handled in the analyses (i.e., scale, units, model) | 2-4 | We used the random-effects inverse variance weighting (IVW) method as the primary analysis to assess the association between circulating cytokines and sepsis risk |
|  | b) | Describe how genetic variants were handled in the analyses and, if applicable, how their weights were selected | 2-4 | All SNPs with a P <5×10-6 were considered significant variants associated with phenotypes and included in order to increase the number of SNPs for further sensitivity analysis and to find potential casual effects |
|  | c) | Describe the MR estimator (e.g. two-stage least squares, Wald ratio) and related statistics. Detail the included covariates and, in case of two-sample MR, whether the same covariate set was used for adjustment in the two samples | 2-4 | We excluded SNPs with R2<0.001 using linkage disequilibrium (LD) analysis. To avoid weak instrument bias. An F-statistic value >10 was regarded as strong enough to avoid weak instrument bias. All IVs in this study had an F-statistic >10 |
|  | d) | Explain how missing data were addressed | 2-4 | Selection of IVs  Statistical Analyses |
|  | e) | If applicable, indicate how multiple testing was addressed | NA | NA |
| 7 | **Assessment of assumptions** | Describe any methods or prior knowledge used to assess the assumptions or justify their validity | 2-4 | An F-statistic value >10 was regarded as strong enough to avoid weak instrument bias. All IVs in this study had an F-statistic >10 |
| 8 | **Sensitivity analyses and additional analyses** | Describe any sensitivity analyses or additional analyses performed (e.g. comparison of effect estimates from different approaches, independent replication, bias analytic techniques, validation of instruments, simulations) | 2-4 | We require consistent causal effects across all three methods (IVW ,weighted median and MR-Egger ) and P < 0.05 to define a significant causal effect.The heterogeneity of the instrument variable was determined by Cochran's Q statistic |
| 9 | **Software and pre-registration** |  | 2-4 | Statistical Analyses |
|  | a) | Name statistical software and package(s), including version and settings used | 2-4 | All MR analyses were performed using R (version 4.1.1, Vienna, Austria) with the TwoSampleMR package (version 0.5.6) |
|  | b) | State whether the study protocol and details were pre-registered (as well as when and where) | NA | NA |
|  | **RESULTS** |  |  |  |
| 10 | **Descriptive data** |  |  |  |
|  | a) | Report the numbers of individuals at each stage of included studies and reasons for exclusion. Consider use of a flow diagram |  | Detailed information can be found in supplementary material table S1 |
|  | b) | Report summary statistics for phenotypic exposure(s), outcome(s), and other relevant variables (e.g. means, SDs, proportions) |  | Detailed information can be found in supplementary material table S2 |
|  | c) | If the data sources include meta-analyses of previous studies, provide the assessments of heterogeneity across these studies |  | NA |
|  | d) | For two-sample MR:  i.  Provide justification of the similarity of the genetic variant-exposure associations between the exposure and outcome samples  ii.  Provide information on the number of individuals who overlap between the exposure and outcome studies |  | Detailed information can be found in supplementary material table S2/S3 |
| 11 | **Main results** |  |  |  |
|  | a) | Report the associations between genetic variant and exposure, and between genetic variant and outcome, preferably on an interpretable scale | 4-5 | Detailed information can be found in Table1 |
|  | b) | Report MR estimates of the relationship between exposure and outcome, and the measures of uncertainty from the MR analysis, on an interpretable scale, such as odds ratio or relative risk per SD difference | 4-5 | Detailed information can be found in Table1 |
|  | c) | If relevant, consider translating estimates of relative risk into absolute risk for a meaningful time period | 4-5 | NA |
|  | d) | Consider plots to visualize results (e.g. forest plot, scatterplot of associations between genetic variants and outcome versus between genetic variants and exposure) | 4-5 | Detailed information can be found in figure 2 |
| 12 | **Assessment of assumptions** |  |  |  |
|  | a) | Report the assessment of the validity of the assumptions | 4-5 | This paper reports the results of the assessment of the validity of the associated hypotheses in several places throughout the text and in the supplementary materials, e.g., Table1, Table 2 and Table S2 . For example, In Table 2, the heterogeneity of the statistical model was tested with the Q-statistic to assess its stability |
|  | b) | Report any additional statistics (e.g., assessments of heterogeneity across genetic variants, such as *I^2^*, Q statistic or E-value) | 4-5 | the results of the Q-statistic in Table 2 |
| 13 | **Sensitivity analyses and additional analyses** |  |  |  |
|  | a) | Report any sensitivity analyses to assess the robustness of the main results to violations of the assumptions | 4-5 | The results of the MR analysis obtained by the three analytical methods, including the weighted median method, MR-Egger and MR-PRESSO, are presented in Table 1 and Table S2 . |
|  | b) | Report results from other sensitivity analyses or additional analyses |  | The results of leave-one-out analysis was shown in Supplementary figure 2/4/6. |
|  | c) | Report any assessment of direction of causal relationship (e.g., bidirectional MR) | NA | NA |
|  | d) | When relevant, report and compare with estimates from non-MR analyses |  |  |
|  | e) | Consider additional plots to visualize results (e.g., leave-one-out analyses) |  | The results of leave-one-out analysis was shown in Supplementary figure 2/4/6. |
|  | **DISCUSSION** |  |  |  |
| 14 | **Key results** | Summarize key results with reference to study objectives | 7 | We found that elevated beta-nerve growth factor increased the risk of sepsis, while decreased RANTES and Fibroblast growth factor decreased the risk of sepsis. |
| 15 | **Limitations** | Discuss limitations of the study, taking into account the validity of the IV assumptions, other sources of potential bias, and imprecision. Discuss both direction and magnitude of any potential bias and any efforts to address them | 8 | The limitations of the study are discussed at great length in the example essay in terms of the data sources, the three main hypotheses, and the analytical process in detail |
| 16 | **Interpretation** |  |  |  |
|  | a) | Meaning: Give a cautious overall interpretation of results in the context of their limitations and in comparison with other studies | 7-8 | The entry is discussed at maximum length in the example text, which rationalizes the MR results by comparing them to multiple published studies. |
|  | b) | Mechanism: Discuss underlying biological mechanisms that could drive a potential causal relationship between the investigated exposure and the outcome, and whether the gene-environment equivalence assumption is reasonable. Use causal language carefully, clarifying that IV estimates may provide causal effects only under certain assumptions | 7-8 | This study discussed the biological mechanism, for example, FGF5 protects against myocardial injury caused by sepsis by inhibiting CaMKII/NF-κB signaling |
|  | c) | Clinical relevance: Discuss whether the results have clinical or public policy relevance, and to what extent they inform effect sizes of possible interventions | 7-8 | The example paper reports uncertainty in the degree of pleiotropy of the 437 SNPs, and therefore uncertainty in the MR results, but still leads to the conclusion that |
| 17 | **Generalizability** | Discuss the generalizability of the study results (a) to other populations, (b) across other exposure periods/timings, and (c) across other levels of exposure | 7-8 | our data source is mainly European population, which makes our findings not directly applicable to others |
|  | **OTHER INFORMATION** |  |  |  |
| 18 | **Funding** | Describe sources of funding and the role of funders in the present study and, if applicable, sources of funding for the databases and original study or studies on which the present study is based |  | The authors received no funding for this work. |
| 19 | **Data and data sharing** | Provide the data used to perform all analyses or report where and how the data can be accessed, and reference these sources in the article. Provide the statistical code needed to reproduce the results in the article, or report whether the code is publicly accessible and if so, where |  | This study provides access to all the information in the beginning of the methodology section. <https://gwasmrcieu.ac.uk/> details can be found in supplementary Table 1 |
| 20 | **Conflicts of Interest** | All authors should declare all potential conflicts of interest |  | The authors declare that they have no competing interests. |

This checklist is copyrighted by the Equator Network under the Creative Commons Attribution 3.0 Unported (CC BY 3.0) license.

1. Skrivankova VW, Richmond RC, Woolf BAR, Yarmolinsky J, Davies NM, Swanson SA, et al. Strengthening the Reporting of Observational Studies in Epidemiology using Mendelian Randomization (STROBE-MR) Statement. JAMA. 2021;under review.

2. Skrivankova VW, Richmond RC, Woolf BAR, Davies NM, Swanson SA, VanderWeele TJ, et al. Strengthening the Reporting of Observational Studies in Epidemiology using Mendelian Randomisation (STROBE-MR): Explanation and Elaboration. BMJ. 2021;375:n2233.
